# Method for the estimation of institutional quality indexes using fuzzy logic

**DOI:** 10.1016/j.mex.2022.101676

**Published:** 2022-03-25

**Authors:** Vinícius Souza Ribeiro

**Affiliations:** Department of Natural Resources, Federal Institute of Education, Science and Technology of Tocantins (IFTO), Palmas, Tocantins, Brazil

**Keywords:** Aquaculture, Fuzzy inference system, Indices, Institutional environment

## Abstract

This paper presents a method to estimate institutional environment indexes using fuzzy modeling. Because of the complexity of the subject, institution, elements associated with this thinking are difficult to measure and compare. In order to address this problem, this research presents how a fuzzy inference system works and how to create institutional indexes from it. While methods that analyze institutional environments generally use secondary data from countries or regions provided by international organizations, the illustrative case applied to aquaculture in Brazil demonstrates the effectiveness of using this method to generate indexes related to the subject from primary data collected at the firm level. Furthermore, the combined use of this method with others already used in the institutional literature can be valuable both for researchers and public policy makers who seek to increasingly understand the role of institutions in economic performance.

• Uses a Mamdani expert system of MIMO type to estimate institutional indexes.
• Institutional ambient scores related to tilapia production in Brazil are presented.
• The combined use of the method with others can be valuable for the research field.

## Graphical abstract

**Figure:**
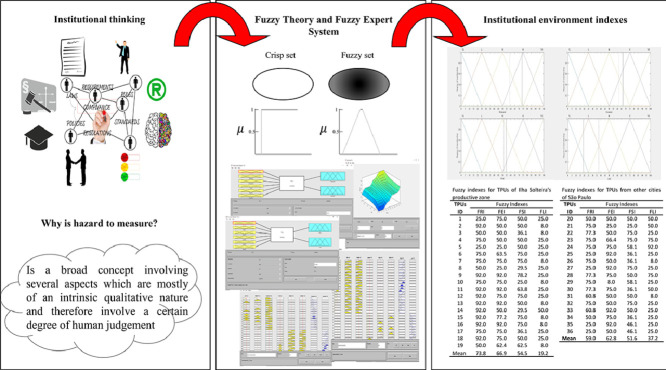

Specifications Table

**Table:** 

Subject Area;	Economics and Finance
More specific subject area;	*Institutions*
Method name;	*Estimation of fuzzy institutional indexes*
Name and reference of original method;	*Mamdani fuzzy inference system* *Mamdani, E.H., 1977. Application of Fuzzy Logic to Approximate Reasoning Using Linguistic Synthesis. IEEE Transactions on Computers, C-26(12), 1182–1191.*https://doi.org/10.1109/TC.1977.1674779
Resource availability;	*MATLAB®*

## Method overview

Institutions have broad concepts that render them difficult to define and are used by several research fields, such as economics, philosophy, sociology, politics, and geography. Even today, its complexity provides conflicting definitions [1]. The literature reveals a list of analytical elements that are difficult to measure or compare given their intrinsic qualitative nature and, therefore, involve a certain degree of human judgment, for example, quality of policies and laws, contract and property rights enforcement, norms, customs, tradition, power, leadership, trust, reciprocity, and access and availability of resources [2].

In order to address this problem, this study presents a novel method for estimating institutional environment indexes using fuzzy modeling in four perspectives (relational, economic, social, and local) applied in an illustrative case of Brazilian aquaculture. This method allows modeling different indicators and indexes that can be used in conjunction with other methods to help measure the role of institutions in economic performance.

As demonstrated in the case applied, this model has the particular ability to process data and information collected at the firm level (micro-level). This expands the field of research of institutions beyond the usual institutional analyses that use secondary data (usually provided by international organizations) and are oriented to analysis at the level of countries and regions [3], [4], [5]. Parallel to this, the use of fuzzy systems in this field can also be significantly useful to (re)shape the proxies used as inputs in econometric models such valuable and widely applied to measure the impact of institutions on economy [6], [7], [8], [9].

## Materials and methods

### Database and study area

**Fig. 1:**
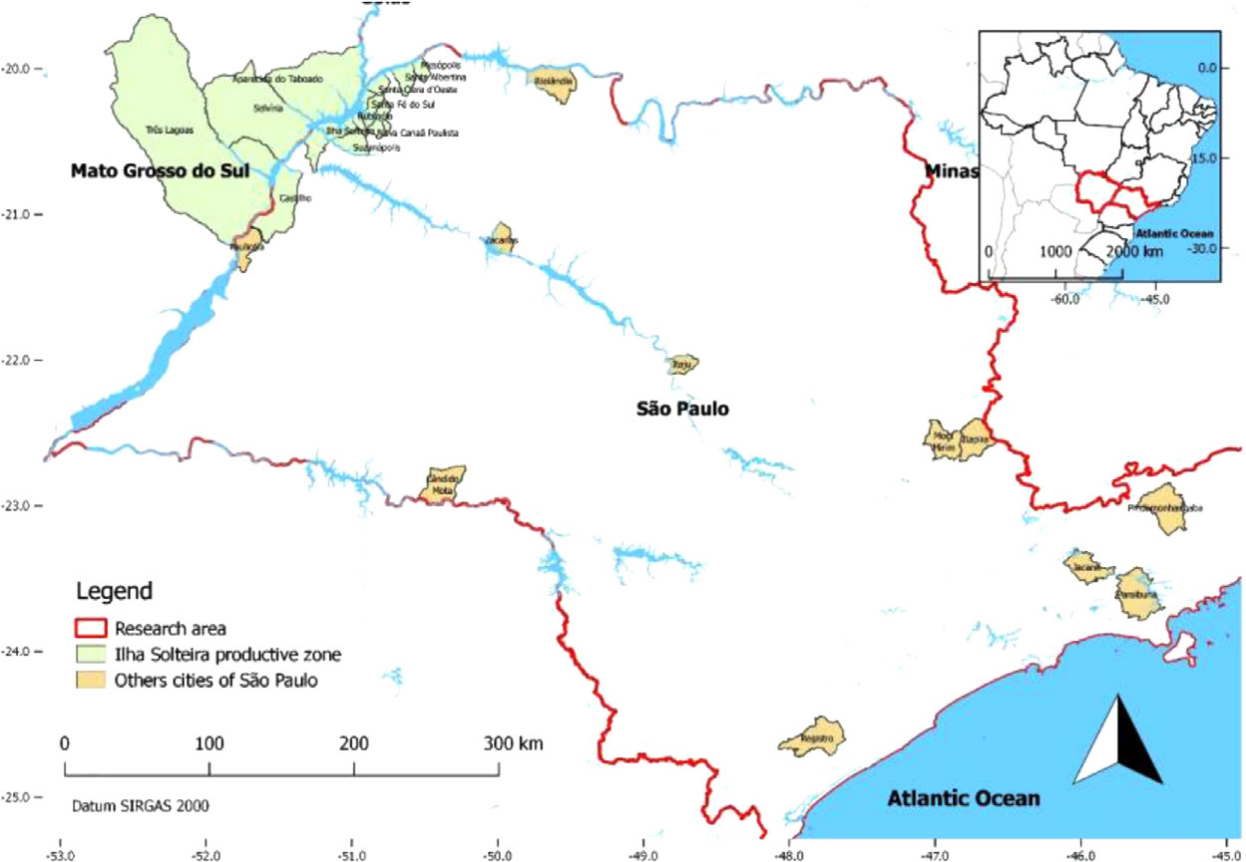
Research spatial distribution. Indicates the cities where the interviewed TPUs are located.

The data were collected in Brazil, in the first quarter of 2019, through a survey directly answered by tilapia production units (TPUs), in the states of São Paulo (SP) and Mato Grosso do Sul (MS). In total, 36 TPUs answered the questionnaire, 19 of them delimited to the productive zone of Ilha Solteira (SP/MS) and the remaining 17 in 11 other cities of São Paulo; therefore the data were tabulated and processed as two different groups: Ilha Solteira and others of São Paulo (Fig. 1).

Due to a methodological issue related to the size of the system (which will be presented further), we have defined four contexts of institutional analysis in this research: (1) Relational, (2) Economic, (3) Social and (4) Local. The first was based on one of the three dimensions of social capital, which applied to the scope of firms proposed by Tsai and Ghoshal [10].^1^ Thus, we seek to insert in the model, more directly, relational aspects inherently immersed in the institutional field. The other three contexts were based on the synthetic parameters of the ‘Local Institutional Context’ analytical dimension proposed by Gereffi and Fernández-Stark [11] for the analysis of Global Value Chains (GVC), and also in the Miranda and Wagner [12], Dollar et al. [13] Dollar and Kidder [14] studies. Table 1 presents these four contexts, with their respective indicators described.

**Table 1:** Contexts and indicators for institutional environment fuzzy indexes.

Context	Indicators	FIS Inputs	Description
Relational	Trust Scale	Conf.	Level of confidence of the producer in its trading partners
	Frequency of change of business partners	FMudPar.	Frequency scale of change business partners by the producer
	Compliance with trade deals	CumpAcord.	Occurrence of non-compliance with trade agreements, characterized by the evaluation of default as a difficulty in commercialization of the product
			
Economic	Quality of infrastructure	Infra.	Evaluation of the quality of roads in the region
	Availability of financial resources	DispRec.	Existence or not of banks or other institutions (development agencies, credit unions, etc.) that offer financing for the activity
	Access to financial resources	AcesRec.	Occurrence or not of factor that restricts access to market credit, when available in the region (i.e. non-compliance with laws)
			
Social	Manager's level of education	NivEsc.	Education level of the TPU's manager
	Women's participation in management	Mulh.	Occurrence or not of women occupying the position of manager
	Availability of qualified workforce	DispMO.	Evaluation of impact level of the availability of skilled labor as a barrier to activity
			
Local	Environmental Legislation	LegAmb.	Evaluation of impact level of existing environmental legislation as a barrier to activity
	Operational Legislation	LegOP.	Evaluation of impact level of the process to obtain the cession of union water use as a barrier to activity
	Public Safety	SegPub	Evaluation of impact level of public safety as a barrier to activity

Regarding the complexity of the subject institutions, the use of four contexts and twelve indicators described in this research do not seek to delimit everything that surrounds this phenomenon, but rather to point out practical definitions for empirical analysis in aquaculture.

### Method details: The configuration and functioning of the fuzzy inference system

The Iranian mathematician Lofti Asker Zadeh with the publication of the article “Fuzzy Sets” first introduced the fuzzy sets theory in 1965. In this, the mathematician sought to solve the problem of the imprecision of the human mind. By Zadeh's theoretical proposition, a fuzzy "A" set defined in the universe of "X" discourse is characterized by a membership function, which maps the elements of X to the interval [0,1] [15].

**Fig. 2:**
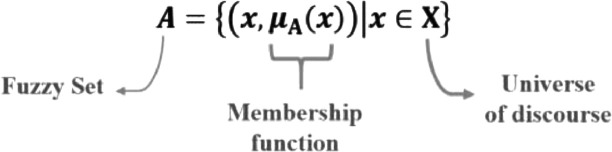
Determination of fuzzy sets.

Thus, the membership function associated with each "x" element belonging to "X is an real-number in the interval [0,1] that represents the degree of membership (adherence) of the "x" element to the set "A" for each (Fig. 2).

Fuzzy set theory is used by fuzzy logic to extend traditional bi-value logic (true or false, yes or no, and so forth) assuming that the true values are nebulous sets defined in the range 0–1. What makes fuzzy logic powerful is its ability to model human thought, since it uses approximate reasoning instead of precise reasoning. This logic provides a significant contribution to research using unclear data, often expressed in linguistic terms, and quite close to human perception [16].

In recent decades, fuzzy logic and fuzzy systems have been widely adopted in the scientific community, since they are input-output models where input variables do not have exact values, but present influential probabilities of each variable on the final outcome [17].

A Fuzzy Inference System (FIS) is a system that maps inputs and processes them based on pre-established rules producing outputs. The architecture of this system can divide into four key elements: fuzzification, rules base, fuzzy inference and defuzzification [18].

Synthetically a FIS can also be understood as an inference process based on fuzzy rules (or approximate reasoning) of the "IF-THEN" type, which connects antecedents and consequents, making use of membership functions and fuzzy operators [19].

**Fig. 3:**
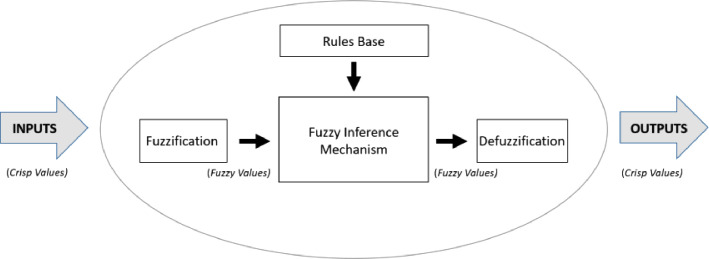
Main elements of Fuzzy Inference System.

Fig. 3 represents the main elements of a FIS. In the fuzzing stage, crisp values translated into fuzzy values, which are determined by the degrees of adherence to the membership functions that are in turn connected to the linguistic variables. The rule base is a structure of linguistic statements of the type "IF-THEN" that stocks all the knowledge of the system. In the inference module, the fuzzy values obtained and processed by inference methods, where operations of implication, composition and aggregation of rules occur. Finally, defuzzification transforms, through different techniques, the aggregation results of the previous step into crisp values [20,21].

**Fig. 4:**
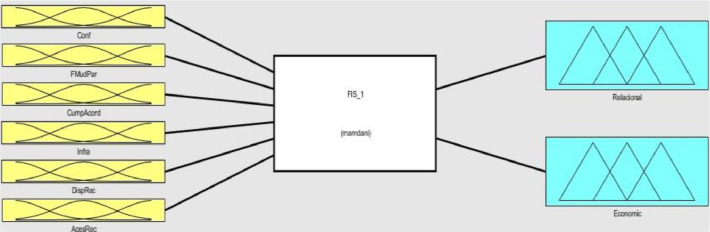
Fuzzy Inference System 1 (FIS 1).

**Fig. 5:**
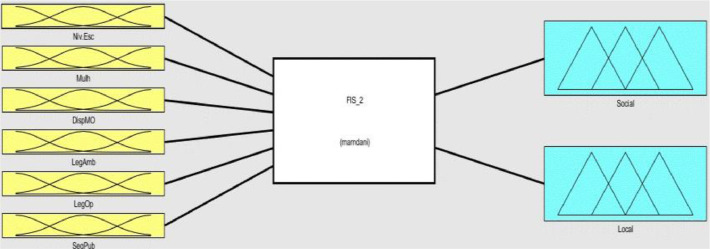
Fuzzy Inference System 2 (FIS 1).

For research purposes, two fuzzy expert systems were implemented with the support of MATLAB® R2018a software. The fuzzy expert system is the most popular of the knowledge-based systems, where knowledge is described by a set of instructions, for example, IF-THEN rules. IF-THEN rule systems are most widely used in the processing and representing fuzzy knowledge [22].

Each one of the two FIS contains six inputs and two outputs, FIS 1 is composed of the inputs relating to the relational and economic context (Fig. 4), and the FIS 2 of the inputs relating to the social and local context (Fig. 5). The two systems generate four outputs that represent indexes of the institutional environment of tilapia farming in their respective contexts.

As previously mentioned, the choice of using four analytical contexts for the institutional environment was due to operational issues of model size. If we chose to use in a single FIS all twelve inputs (without division into contexts) with a single output (representing a single institutional environment index) the total of rules at the base of the system would be enormous, with more than 194,000 rules. This methodological alternative of fuzzy sub-indexes using two FIS also facilitates the operational work of inserting a large number of rules into the software.

In this research, given the characteristics of the indicators and the linguistic variables associated with them, the FIS inputs have taken on triangular and singleton membership function (MF) forms. The outputs took on strictly triangular functions. Eqs. (1) and 2 represent these two kinds of MFs, with parameters (a,m,b), being *a* ≤ *m* ≤ b*,* with a, m, b and x belonging to the universe set U.

(1) $$\text{Triangular}:\;\mu_{A}\left(X\right)=\left\{\begin{array}{c} 0,\;x<a\\ \frac{\left(x-a\right)}{\left(m-a\right)},\;a\leq x\leq m\\ \frac{\left(b-x\right)}{\left(b-m\right)},\;m\;\leq x\leq b\\ 0,\;x>b \end{array}\right\}$$

(2) $$Singleton:\;\mu_{A}\left(X\right)\;=\left\{ \begin{array}{c} 1,\;if\;x=m\\ 0,\;otherwise \end{array}\right.$$

According to the characteristics of each input and output of the systems, the functions connected to the linguistic terms are: Very Low (VL), Low (L), Medium (M), High (H) and Very High (VH). The transformation of the previous and consequent linguistic terms into fuzzy numbers through the MFs are represented in Tables 2 and 3, respectively.

**Table 2:** Transformation of antecedent linguistic terms into fuzzy numbers.

Terms	Fuzzy Numbers		
	5 Terms	3 Terms	2 Terms
Very Low	(0, 0, 0.25)	———	———
Low	(0, 0.25, 0.5)	(0, 0, 0.5)	(0)
Medium	(0.25, 0.5, 0.75)	(0, 0.5, 1)	———
High	(0.5, 0.75, 1)	(0.5, 1, 1)	(1)
Very High	(0.75, 1, 1)	———	———

**Table 3:** Transformation of consequent linguistic terms into fuzzy numbers.

Terms	Fuzzy Numbers
Very Low	(0, 0, 25)
Low	(0, 25, 50)
Medium	(25, 50, 75)
High	(50, 75, 100)
Very High	(75, 100, 100)

Rules bases of MIMO (Multiple-Input/Multiple-Output) type were used, composed by IF-THEN rules, having a previous part (premise) and consequent part (conclusion) connected by the logical connective (operator) “AND”, as shown in Table 4.

**Table 4:** Inference rules for relational context.

Rule Number	Inference Rules
1	IF < (Conf is H) *and* (FMudPar is H) *and* (CumpAcord is H) > THEN < (Relational VH)>
2	IF < (Conf is H) *and* (FMudPar is H) *and* (CumpAcord is L) > THEN < (Relational H)>
3	IF < (Conf is H) *and* (FMudPar is L) *and* (CumpAcord is H) > THEN < (Relational H)>
4	IF < (Conf is L) *and* (FMudPar is H) *and* (CumpAcord is H) > THEN < (Relational H)>
5	IF < (Conf is M) *and* (FMudPar is H) *and* (CumpAcord is H) > THEN < (Relational H)>
6	IF < (Conf is H) *and* (FMudPar is M) *and* (CumpAcord is H) > THEN < (Relational H)>
7	IF < (Conf is M) *and* (FMudPar is H) *and* (CumpAcord is L) > THEN < (Relational M)>
8	IF < (Conf is H) *and* (FMudPar is M) *and* (CumpAcord is L) > THEN < (Relational M)>
9	IF < (Conf is M) *and* (FMudPar is M) *and* (CumpAcord is L) > THEN < (Relational M)>
10	IF < (Conf is M) *and* (FMudPar is L) *and* (CumpAcord is H) > THEN < (Relational M)>
11	IF < (Conf is L) *and* (FMudPar is M) *and* (CumpAcord is H) > THEN < (Relational M)>
12	IF < (Conf is M) *and* (FMudPar is M) *and* (CumpAcord is H) > THEN < (Relational M)>
13	IF < (Conf is L) *and* (FMudPar is L) *and* (CumpAcord is H) > THEN < (Relational L)>
14	IF < (Conf is H) *and* (FMudPar is L) *and* (CumpAcord is L) > THEN < (Relational L)>
15	IF < (Conf is L) *and* (FMudPar is H) *and* (CumpAcord is L) > THEN < (Relational L)>
16	IF < (Conf is M) *and* (FMudPar is L) *and* (CumpAcord is L) > THEN < (Relational L)>
17	IF < (Conf is L) *and* (FMudPar is M) *and* (CumpAcord is L) > THEN < (Relational L)>
18	IF < (Conf is L) *and* (FMudPar is L) *and* (CumpAcord is L) > THEN < (Relational VL)>

*Note:* VL is very low; L is low; M is medium; H is high; VH is very high.

The definition of the rules base, a central part of FIS, was constituted from deductive logical reasoning based on economic analysis and the literature of the social capital [23,24] and transaction cost economics [25]. It is important to highlight that with MATLAB®, it is possible to assign different weights to each of the inserted rules, which gives a greater possibility of customization of the system. No weights were set in this illustrative case, so all the rules had the same importance for the generation of the indexes.

Table 5 shown the general causal mechanism between indicators and institutional environment.

**Table 5:** General causal mechanism between indicators and institutional environment.

Context	Indicators	Limits of institutional environment	
		Worst scenario	Best scenario
Relational	Trust scale	worst rating	best rating
	Frequency of change of business partners	high occurrence	non-occurrence
	Compliance with trade deals	non-occurrence	occurrence
Economic	Quality of infrastructure	worst rating	best rating
	Availability of financial resources	non-occurrence	occurrence
	Access to financial resources	restricted	unrestricted
Social	Manager's level of education	illiterate	post graduate
	Women's participation in management	non-occurrence	occurrence
	Availability of qualified workforce	high impact	non-impact
Local	Environmental legislation	high impact	non-impact
	Operational legislation	high impact	non-impact
	Public safety	high impact	non-impact

*Note:* The logic of relationship between indicators and the quality of the institutional environment was guided, in addition to the reinforcing of gender equality (female managers), by the economic perspectives of increasing/reducing of transaction costs, labor productivity and barriers to entry and exit.

In all, 85 rules constituted 38 for FIS 1 and 47 for FIS 2. In more detail, for the Fuzzy Index of Institutional Environment in the Relational Context (FRI) rules were constructed referring to the respective linguistic terms (L, M, H) of each of the three indicators, totalling 18 (3 × 3 × 2) rules. For the Economic Context Index (FEI), 20 (5 × 2 × 2); Social (FSI), 20 (5 × 2 × 2) and Local (FLI), 27 (3 × 3 × 3). Table 4 presents the rules used for FRI in FIS 1, while Table 6 summarizes the 85 rules used for both FIS.

**Table 6:** Summary of rules used for both FIS.

Relational		Economic		Social		Local	
IF	THEN	IF	THEN	IF	THEN	IF	THEN
H-H-H	VH	VH-H-H	VH	VH-H-H	VH	H-H-H	VH
H-H-L	H	H-H-H	VH	H-H-H	VH	L-H-H	H
L-H-H	H	VH-L-H	H	VH-L-H	H	M-H-H	H
H-L-H	H	VH-H-L	H	VH-H-L	H	H-L-H	H
M-H-H	H	H-L-H	H	H-L-H	H	H-M-H	H
H-M-H	H	H-H-L	H	H-H-L	H	H-H-L	H
M-H-L	M	M-H-H	H	M-H-H	H	H-H-M	H
H-M-L	M	L-H-H	H	L-H-H	H	L-M-M	M
M-M-L	M	VH-L-L	M	VH-L-L	M	L-M-H	M
M-L-H	M	M-L-H	M	M-L-H	M	L-H-M	M
L-M-H	M	M-H-L	M	M-H-L	M	M-L-M	M
M-M-H	M	VL-H-H	M	VL-H-H	M	M-L-H	M
L-L-H	L	H-L-L	L	H-L-L	L	M-M-L	M
H-L-L	L	M-L-L	L	M-L-L	L	M-M-M	M
L-H-L	L	L-L-H	L	L-L-H	L	M-M-H	M
M-L-L	L	L-H-L	L	L-H-L	L	M-H-L	M
L-M-L	L	VL-L-H	L	VL-L-H	L	M-H-M	M
L-L-L	VL	VL-H-L	L	VL-H-L	L	H-L-M	M
		L-L-L	VL	L-L-L	VL	H-M-L	M
		VL-L-L	VL	VL-L-L	VL	H-M-M	M
						L-L-M	L
						L-L-H	L
						L-M-L	L
						L-H-L	L
						M-L-L	L
						H-L-L	L
						L-L-L	VL
Total of Rules	18		20		20		27

*Note:* VL is very low; L is low; M is medium; H is high; VH is very high.

When using rule bases such as those described above, conclusions should be based on all rules, thus making it necessary to aggregate all individual relationships into one set of rules. For this aggregation, several methods are used. However, most methods applied are Geramian et al. [20].

Although it is possible to apply either method through the MATLAB® software, the Mamdani [26] method was chosen for this illustrative case, for two reasons. Firstly, the fuzzy output of the system is accompanied by the membership function, which for this article leaves the presentation of results more intuitive, in the method of Sugeno this does not happen. The second factor is that the chosen method supports MIMO watering systems, while the other only MISO (Multiple-Input/Single-Output)^2^.

When there are multiple precedents applied to a rule, the fuzzy operator (i.e., AND, OR, NOT) is used to obtain a single number that represents the result of the previous evaluation. To evaluate the disjunction (intersection) of the rule of precedent, the fuzzy operation "AND" is used, in an analogous way for conjunction (union), the operator "OR" is used, while for complement (complement) the operator is "NOT" [27]. These are expressed by the operations, where s is a S-norm and t is a T-norm:

(3) $$\mu_{A\cap B}\left(x\right)=s\left\{\mu_{A}\left(x\right),\mu_{B}\left(x\right)\right\}=min\left\{\mu_{A}\left(x\right),\mu_{B}\left(x\right)\right\}\;\left(intersection\right)$$

(4) $$\mu_{A\cup B}\left(x\right)=t\left\{\mu_{A}\left(x\right),\mu_{B}\left(x\right)\right\}=max\left\{\mu_{A}\left(x\right),\mu_{B}\left(x\right)\right\}\;\left(union\right)$$

(5) $$\mu_{co\left(A\right)}\;\left(x\right)=\;1-\;\mu_{A}\left(x\right)\;\left(complement\right)$$

The logical operations are formed such that the function *min* and function *max* are among the most applied. Although other functions such as product and probabilistic OR are also applicable in the expression of these fuzzy operators, function *min* and function *max* are always simple, effective and widely used [28].

Taking as a rule, of the type:

(6) $$\text{IF}\;x\;\text{is}\;A_{i}\;\text{THEN}\;y\;\text{is}\;B_{i},\;i=1,\;\ldots ,\;n$$

In Mamdani's model, the outputs^3^ are built by the superposition of the consequent individual rules, of the type [29]:

$$R_{1\;}:\;\text{IF}\;x_{1}\;\text{is}\;A_{1}\;\text{THEN}\;y_{1}\;\text{is}\;B_{1}$$

$$R_{2\;}:\;\text{IF}\;x_{2}\;\text{is}\;A_{2}\;\text{THEN}\;y_{2}\;\text{is}\;B_{2}$$...;

(7) $$R_{i\;}:\;\text{IF}\;x_{i}\;\text{is}\;A_{i}\;\text{THEN}\;y_{i}\;\text{is}\;B_{i}$$

Being, $A_{i}$ e $B_{i}$ fuzzy subsets of universes *U* e *V*, each rule can be interpreted from as:

(8) $$R_{i\;}=\;A_{i}⊗B_{i}$$

Where, ⊗ is product operation. Being their membership function given by:

(9) $$\mu_{\text{Ri}}\left(x,y\right)=\min\left(\mu_{Ai}\left(x\right),\;\mu_{Bi}\left(y\right)\right)$$

In summary, according to each rule (8) control of a system (6), the method of Mamdani used all individual rules to compose them into fuzzy *R* relationship, to the whole system. Being the aggregation operator represented by "⋃", we have [29]:

(10) $$R={⋃}_{i=1}^{n}R_{i}={⋃}_{i=1}^{n}\left(A_{i}⊗B_{i}\right)$$

With membership function $\;\mu_{R}\left(x,y\right)$:

(11) $${⋃}_{i=1}^{n}R_{i}\left(x,y\right)=max\left\{\min(\mu_{Ai}\left(x\right),\;\mu_{Bi}\left(y\right)\right\}$$

Based on the compositional inference rule suggested by Zadeh [30], we have the output set B´(*y*):

(12) $$B^{\prime }\left(y\right)=A^{\prime }\left(x\right)\circ R\left(x,y\right)$$

Being, “∘” a compositional operator, whose membership function is given by:

(13) $$\mu_{B}\left(y\right)$$

Where, ∧  is a t-norm operator.sss

After the inference module, defuzzification occurs, which consists in determining the crisp value, that is, obtaining the best representation for the fuzzy output set, applying a defuzzification method to the set $B^{\prime }={⋃}_{\left(i=1\right)}^{k}{B^{\prime }}_{i}$ resulting from the aggregation of all fuzzy output sets $B_{i}^{\prime },\;i=1,2,\;\ldots ,\;k$. This way, defuzzification consists in converting the outputs of fuzzy rules into a crisp value, through different schemes, being the main ones: center of area (CoA), center of gravity (CoG) and the mean of maxima (MoM) [22].

In this illustrative case, the CoA method was used, also known as centroid. This method determines the center of the fuzzy set area and returns the corresponding crisp value. The center of the area is calculated by:

(14) $$CoA=\;\frac{\sum_{k=1}^{n}\mu_{A\;}\left(X_{k}\right)\;X_{k}}{\sum_{k=1}^{n}\mu_{A\;}\left(X_{k}\right)}$$

Finally, the fuzzy indexes of the institutional environment for each context are the outputs of the FISs for each TPU. Bearing in mind that their values are defined in a range from 0 to 100, where 100 is considered the best possible institutional environment; conversely, given the context of analysis, and analogously 0 is regarded as the worst.

## Results

**Fig. 6:**
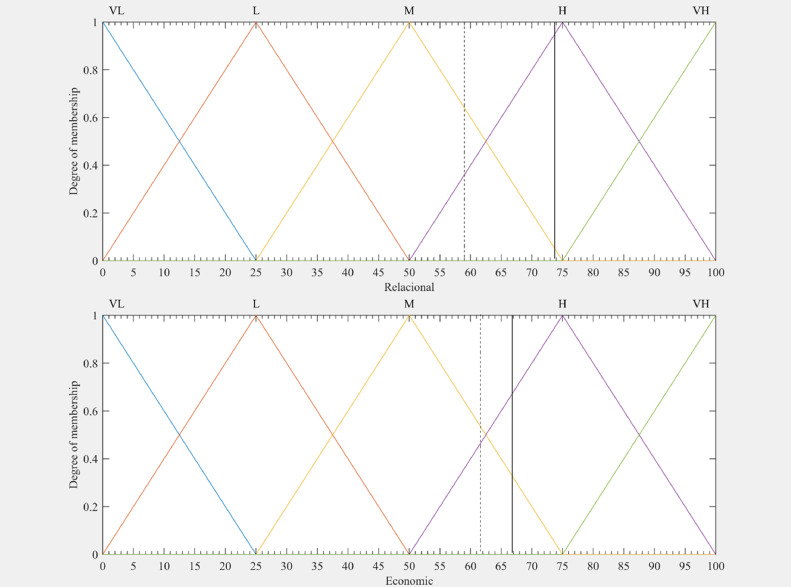
Outputs of inference systems with the membership functions for both groups. Legend: Ilha Solteira's productive zone (continuous line) and Group of other cities of São Paulo (dashed line); VL is very low; L is low; M is medium; H is high; VH is very high.

**Fig. 7:**
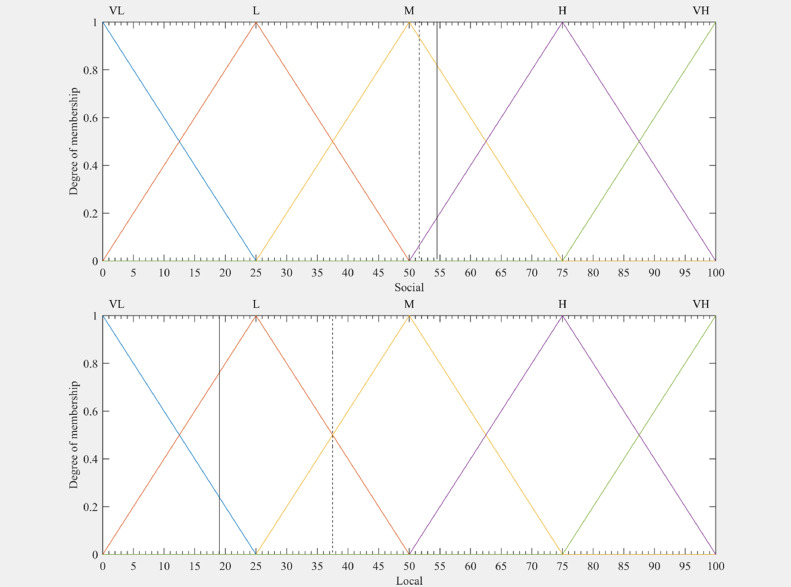
Social and local contexts: outputs of inference systems with the membership functions for both groups. Legend: Ilha Solteira's productive zone (continuous line) and Group of other cities of São Paulo (dashed line); VL is very low; L is low; M is medium; H is high; VH is very high.

Tables 7 and 8, as well as Figs. 6 and 7, present the results of the research and validate the use of the method in the task of estimating institutional environment indexes.

**Table 7:** Fuzzy indexes for TPUs of Ilha Solteira's productive zone.

TPUs ID	Fuzzy Indexes			
	FRI	FEI	FSI	FLI
1	25.0	75.0	50.0	25.0
2	92.0	50.0	50.0	8.0
3	50.0	50.0	36.1	8.0
4	75.0	50.0	50.0	25.0
5	25.0	25.0	50.0	25.0
6	75.0	63.5	75.0	25.0
7	75.0	75.0	75.0	8.0
8	50.0	25.0	29.5	25.0
9	92.0	92.0	78.2	25.0
10	75.0	75.0	25.0	8.0
11	92.0	92.0	63.8	25.0
12	92.0	75.0	75.0	25.0
13	92.0	92.0	50.0	8.0
14	92.0	50.0	29.5	50.0
15	92.0	77.2	75.0	8.0
16	92.0	92.0	75.0	8.0
17	75.0	75.0	36.1	25.0
18	92.0	75.0	50.0	25.0
19	50.0	62.4	62.5	8.0
Mean	**73.8**	**66.9**	**54.5**	**19.2**

*Note:* TPUs ID is tilapia production unit's identification; FRI is fuzzy relational index; FEI is fuzzy economic index; FSI is fuzzy social index; FLI is fuzzy local index.

**Table 8:** Fuzzy indexes for TPUs from other cities of São Paulo.

TPUs ID	Fuzzy Indexes			
	FRI	FEI	FSI	FLI
20	50.0	50.0	50.0	50.0
21	75.0	25.0	25.0	50.0
22	77.3	50.0	75.0	25.0
23	75.0	66.4	75.0	75.0
24	75.0	75.0	58.1	92.0
25	25.0	92.0	36.1	25.0
26	75.0	50.0	36.1	8.0
27	25.0	92.0	75.0	25.0
28	77.3	75.0	50.0	75.0
29	75.0	8.0	58.1	25.0
30	77.3	75.0	36.1	50.0
31	60.8	50.0	50.0	8.0
32	75.0	50.0	75.0	25.0
33	60.8	92.0	50.0	25.0
34	50.0	75.0	36.1	25.0
35	25.0	92.0	46.1	25.0
36	25.0	50.0	46.1	25.0
Mean	**59.0**	**62.8**	**51.6**	**37.2**

*Note:* TPUs ID is tilapia production unit's identification; FRI is fuzzy relational index; FEI is fuzzy economic index; FSI is fuzzy social index; FLI is fuzzy local index.

Some brief analysis can be made from the FIS outputs. In the relational context, there is a better institutional condition of the companies of the Ilha Solteira group (73.8) compared to the other group (59.0). The condition of a High (Fig. 6) relational institutional environment for the Ilha Solteira TPU's was due to better performance of the indicators of frequency of change of trading partners (FMudPar.) and compliance with trade agreements (CumpAcord.).

The FEI and FSI indexes were not statistically different between the groups, with levels between Medium and High for both. On the other hand, in the local context, the FLI of the other TPU's in the state of São Paulo (37.2) indicated a better institutional environment (between Medium and Low) compared to that of Ilha Solteira (19.2) with an environment evaluated between Low and Very Low, as shown in Fig. 7. It is important to note that this context was the most critical among the four estimated for evaluating the institutional environment of fish farms, mainly because of the level of environmental (LegAmb) and operational (LegOP) legislation indicators.

In General, environmental and water cession legislation were the key issues for this critical environment. Despite the difference between the two groups, in broad terms both understood these legislations as barriers, which would lead to a significant impact on the activity. The Compliance with environmental regulations and water cession legislation are recognized bottlenecks in the chain, as pointed out in studies from other Brazilian regions such as Ribeiro [31], Pedroza Filho et al. [32], Valenti et al. [33], Ribeiro and Pedroza Filho [34].

## Conclusions

The article showed how a fuzzy inference system works and how to estimate institutional indexes based on fuzzy logic. The illustrative case demonstrated how indicators created to evaluate an institutional environment can be processed in an expert model. The fuzzy thinking can be useful to better elucidate how institutional aspects of the most different theoretical schools influence the economic performance in micro-level studies, and it is important to note that the method presented can be used with those already prevalent in the research field. For example, it can be used as a step before the use of econometric models, in order to re(model) its inputs. Nevertheless, it can also stimulate new areas of research for several disciplines that take into account institutional thinking being valuable for researchers and policy makers.

As the fuzzy logic provides a significant contribution to research using unclear data, often expressed in linguistic terms, and quite close to human perception, its possibilities for use are extensive. For example, fuzzy modeling can be useful for social sciences and applied social sciences to analyze issues such as poverty, social capital and upgrading (economic, social or environmental). As well, as studies involving more specific analysis such as governance, performance, risk (credit, production or management) and decision-making processes.

## Conflicts of Interest

The author declares that he has no known competing financial interests or personal relationships that might have appeared to influence the work reported in this article.

## Footnotes

1 For the author, social capital covers several aspects of a social context. In the relational dimension, the properties that sustain relationships, such as trust and reciprocity, are highlighted. The other two interrelated dimensions proposed by the author were Structural and Cognitive.

2 For an illustrative case of comparison between the use of the two methods and a synthesis of the differences between them see Hamam and Georganas [35] and Shleeg and Ellabib [36].

3 The inference of an output fuzzy value given an input value is only possible due to the *generalized modus ponens* rule, see Hellendoorn [37].
